# Treadmill Exercise Induces Neutrophil Recruitment into Muscle Tissue in a Reactive Oxygen Species-Dependent Manner. An Intravital Microscopy Study

**DOI:** 10.1371/journal.pone.0096464

**Published:** 2014-05-05

**Authors:** Albená Nunes-Silva, Priscila T. T. Bernardes, Bárbara M. Rezende, Fernando Lopes, Elisa C. Gomes, Pedro E. Marques, Paulo M. A. Lima, Cândido C. Coimbra, Gustavo B. Menezes, Mauro M. Teixeira, Vanessa Pinho

**Affiliations:** 1 Laboratório de Resolução da Resposta Inflamatória, Instituto de Ciências Biológicas da Universidade Federal de Minas Gerais, Belo Horizonte, Minas Gerais, Brazil; 2 Laboratório de Imunobiofotônica, Departamento de Morfologia, Instituto de Ciências Biológicas da Universidade Federal de Minas Gerais, Belo Horizonte, Minas Gerais, Brazil; 3 Laboratório de Endocrinologia e Metabolismo, Departamento de Fisiologia e Biofísica, Instituto de Ciências Biológicas da Universidade Federal de Minas Gerais, Belo Horizonte, Minas Gerais, Brazil; 4 Laboratório de Imunofarmacologia, Departamento de Bioquímica e Imunologia, Instituto de Ciências Biológicas da Universidade Federal de Minas Gerais, Belo Horizonte, Minas Gerais, Brazil; University of Illinois at Chicago, United States of America

## Abstract

Intense exercise is a physiological stress capable of inducing the interaction of neutrophils with muscle endothelial cells and their transmigration into tissue. Mechanisms driving this physiological inflammatory response are not known. Here, we investigate whether production of reactive oxygen species is relevant for neutrophil interaction with endothelial cells and recruitment into the quadriceps muscle in mice subjected to the treadmill fatiguing exercise protocol. Mice exercised until fatigue by running for 56.3±6.8 min on an electric treadmill. Skeletal muscle was evaluated by intravital microscopy at different time points after exercise, and then removed to assess local oxidative stress and histopathological analysis. We observed an increase in plasma lactate and creatine kinase (CK) concentrations after exercise. The numbers of monocytes, neutrophils, and lymphocytes in blood increased 12 and 24 hours after the exercise. Numbers of rolling and adherent leukocytes increased 3, 6, 12, and 24 hours post-exercise, as assessed by intravital microscopy. Using LysM-eGFP mice and confocal intravital microscopy technology, we show that the number of transmigrating neutrophils increased 12 hours post-exercise. Mutant gp91^phox-/-^ (non-functional NADPH oxidase) mice and mice treated with apocynin showed diminished neutrophil recruitment. SOD treatment promoted further adhesion and transmigration of leukocytes 12 hours after the exercise. These findings confirm our hypothesis that treadmill exercise increases the recruitment of leukocytes to the postcapillary venules, and NADPH oxidase-induced ROS plays an important role in this process.

## Introduction

Intense, unaccustomed, and eccentric exercise is associated with reactive skeletal muscle inflammatory responses [1]–[2]. These types of physical activity result in disruptions in the cytoskeleton and plasma membrane of skeletal muscle cells, which may occur because of the increased mechanical load [2]. This type of muscle damage is also associated with an increase in circulating muscle proteins, such as creatine kinase and myoglobin, and a decrease in motor control [3]. Structural abnormalities in the muscle that are also associated with this type of damage include sarcolemmal disruption, distortion of the myofibrillar component, Z- line streaming, fragmentation of the sarcoplasmic reticulum, lesions in the plasma membrane, changes in the extracellular myofiber matrix, and swollen mitochondria [4], [5].

There is a growing body of evidence that dead cells or cells undergoing various types of physical or chemical stress trigger a potent inflammatory response by releasing necrosis-derived products, including genomic and mitochondrial DNA, formyl-peptides, F-actin, reactive oxygen species (ROS) and others [6]. Once within the interstitium, these molecules may activate resident cells to release chemotactic mediators [6], [7], which may act on leukocytes and make them adhere to, and transmigrate through, the endothelial wall [8].

Release of ROS may occur after trauma-induced muscle cell death [9]. During intense or repeated skeletal muscle contractile activity, which does not cause cell death but intense cell stress, ROS may also be generated through different mechanisms, including nicotinamide adenine dinucleotide phosphate oxidases (NADPH oxidase), electron transport chains (ETC), and xanthine oxidases (XO) [10]. Transient oxidative stress is necessary for the activation of various signal transduction pathways in inflamed muscle cells, but prolonged severe oxidative stress may alter the intracellular antioxidant homeostasis and long-term muscle integrity [11]. Moreover, it has been shown that NADPH oxidase–dependent ROS may contribute to neutrophil migration [12]. However, the precise role that ROS play during the inflammatory response that occurs during and after exercise is unknown. In this study, we investigated the role that ROS play during the leukocyte recruitment that occurs after a fatiguing exercise protocol in mice.

## Materials and Methods

### Experimental protocol

**Figure 1:**
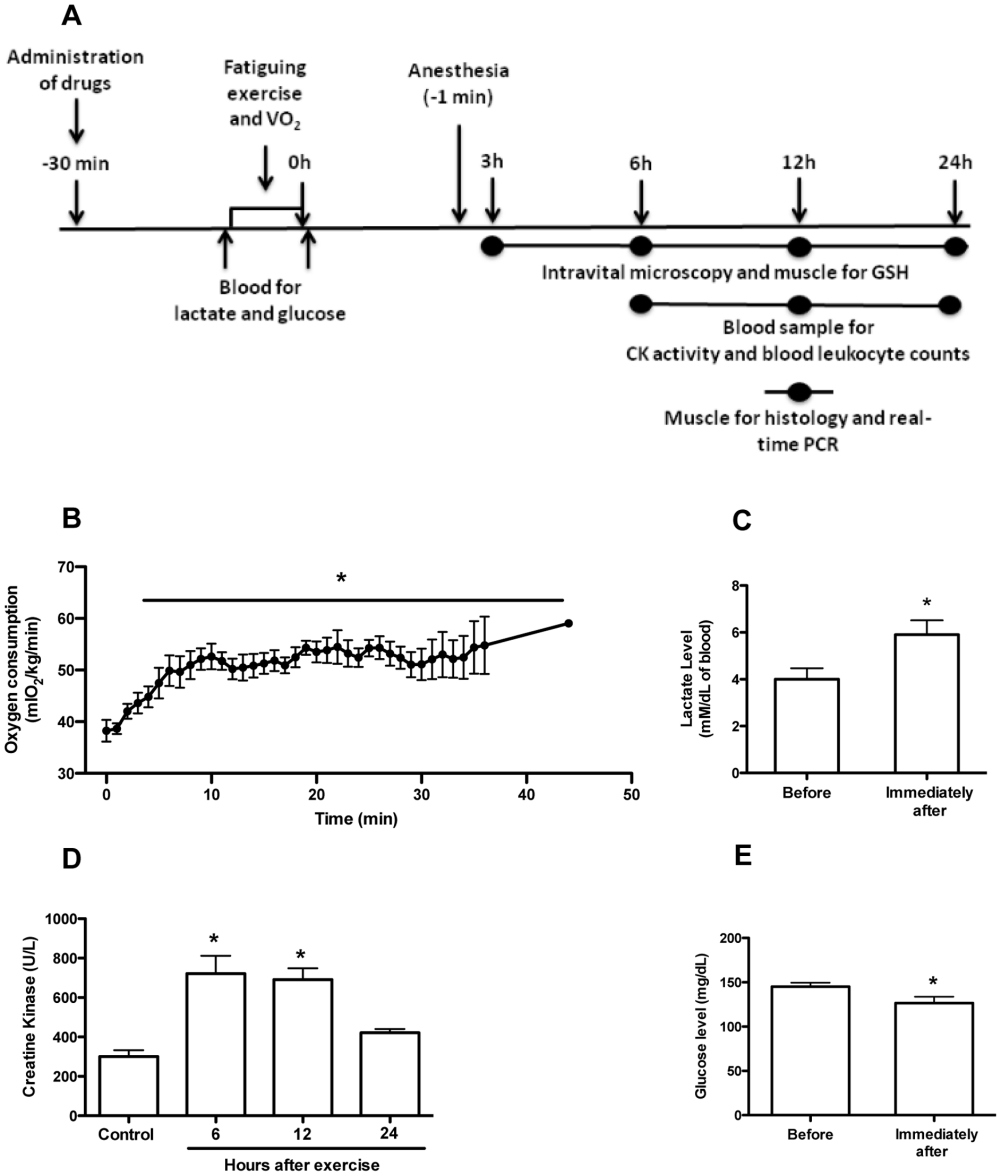
Oxygen consumption, lactate and creatine kinase (CK) levels in the blood. Overall experimental procedures (A). Fatigue was induced by spontaneous running on an electric treadmill that elevated the oxygen consumption (B). At different time-points after the fatiguing exercise blood samples were collected for measurement of lactate (C), creatine kinase (D) and glucose level (E). In this study WT C57Bl/6 mice were used. These results C, D and E are presented as the mean ± SEM (n = 5). *P<0.05 when compared with the control group.

Figure 1A provides a detailed overview of the experimental protocol used in our study. First, animals were subjected to the exercise protocol and oxygen consumption (VO_2_) was measured during time of running. Drug treatment was performed 30 min before the fatiguing exercise protocol. Lactate and glucose were assessed in blood collected immediately before and after the exercise protocol (without anesthesia). At different time-points after exercise, animals were anesthetized and prepared for intravital microscopy, when quadriceps muscle was collected for GSH assay. At 6, 12 and 24h after exercise, blood was collected from the brachial plexus for leukocyte counts and analyses of CK activity. At 12 hour (at the peak of leukocyte accumulation) muscle was collected for histopathological analyses and expression of adhesion molecules by real-time PCR. Finally, we also conducted experiments in mice exercised to 60% of duration or 60% of the intensity to generate a different exercise overload. After intravital microscopy all mice were euthanized by overdose of anesthesia.

### Animals

The male wild-type C57Bl/6 (n = 156), LysM-eGFP C57Bl/6, mice carrying a knock-in mutation for enhanced green fluorescent protein-eGFP-in the lysozyme M-locus (n = 25) and gp91phox-deficient C57Bl/6 mice, non-functional NADPH oxidase (n = 5), weighing 25–30 g were housed in groups of 5 mice per cage at room temperature (25°C) with water and food *ad libitum* and kept on a 12 hours light–12 hours dark cycle. The animal care and handling procedures were in accordance with the guidelines of the Institutional Animal Care and Use Committee, and the study received prior approval from the local animal ethics committee (Animal Ethics Review Board – Comitê de Ética em Experimentação Animal-CETEA/Universidade Federal de Minas Gerais-UFMG, Certificate number 17412/12). The number of mice in each specific group (6 in each group, with the exception of animals not subjected to exercise, n = 4) is provided in the relevant figure legend.

### Fatiguing exercise protocol

An electric treadmill (EP - 132 - INSIGHT) was used for the fatiguing exercise protocol. The initial speed was set at 5 meters per minute (m/min) for 30 minutes to familiarize the mice with the apparatus and task. The speed was then increased 1 m/min every 3 min, at a 5% grade, until the animal stopped running and was fatigued, which was judged by the refusal of the mouse to continue moving on the treadmill belt more than 10 seconds. A mild electrical stimulus (0.5 mA) was applied to mice that stepped off the treadmill to keep them exercising [13]. The control group did not perform fatiguing exercise protocol.

### Protocol at 60% of duration (100% of intensity)

An electric treadmill (EP - 132 - INSIGHT) was used for this protocol. As in the fatiguing exercise protocol, the initial speed was set at 5 meters per minute (m/min) for 30 minutes to familiarize the mice with the apparatus and task. After that, the speed was then increased 1 m/min every 2 min, at a 5% grade, until the animal reached the same maximum velocity of the fatiguing exercise protocol (±15.8 meters/minute). This happened after approximately 36 minutes, after which the test was then interrupted. A continuous, mild electrical stimulus (0.5 mA) was applied to mice that stepped off the treadmill to keep them exercising.

### Protocol at 60% of intensity (100% of duration)

An electric treadmill (EP - 132 - INSIGHT) was used for this protocol. As was done in the fatiguing exercise protocol, the initial speed was set at 5 meters per minute (m/min) for 30 minutes to familiarize the mice with the apparatus and task. The speed was then increased 1 m/min every 3 min until 60% of the maximum intensity of the fatiguing exercise protocol, which is 9 meters/minute, at a 5% grade, until the animals reached the same maximum duration of the fatiguing exercise protocol (±56 minutes). A continuous, mild electrical stimulus (0.5 mA) was applied to all mice that stepped off the treadmill to keep them exercising.

### Oxygen consumption (VO_2_)

On the day of the oxygen consumption (VO_2_) experiment, the animals were allowed to rest for 1 hour in the rodent treadmill chamber before the test. Exercise was performed on a motor driven treadmill (Treadmill Control HARVARD apparatus model LE8710) between 12:00PM and 4:00PM at a room temperature of 22±2°C. Fatigue was defined as the point at which the animals were no longer able to keep pace with the treadmill. Oxygen consumption (VO2) was measured by an open-flow indirect calorimeter (LE 404 Gas Analyzer HARVARD Apparatus) calibrated before each use with a certified mixture of gases (20.5% O2 and 0.5% CO2). VO2 (mlO_2_/ kg/min) was continuously recorded on-line, every minute at rest and during the fatiguing exercise protocol (exercise protocol) and then analysed using a computerized system (Metabolism V 2.2.01) [14]. The data are expressed as mean ± standard error mean (S.E.M.) and the significance level was set at *p*<0.05. The changes in VO2 max. during exercise were tested using one-way analysis of variance (ANOVA) with repeated measures, followed by post hoc Student Newman-Keuls test.

### Lactate and glucose analysis

The lactate and glucose concentration in a drop of blood from the tail vein was analyzed immediately before and after the fatiguing exercise protocol, the protocol at 60% duration, and the protocol at 60% intensity using a lactimeter Accutrend Plus Roche [15].

### Creatine Kinase (CK-NAC)

Creatine kinase (CK-NAC) activity was evaluated using a commercial kit (Biotecnica, Brazil) and a Shimadzu (UV-160A UV) spectrophotometer using blood sample collected from anesthetized mice after exercise protocol at the indicated times (6, 12 and 24 h after exercise). Briefly, plasma CK catalyzes the phosphorylation reaction of ADP in the presence of creatine phosphate to form creatine and ATP. ATP reacts with glucose in the presence of hexokinase, forming ADP and glucose-6-phosphate (G6P), which, in the presence of glucose-6-phosphate dehydrogenase (G6-P-DH), produces 6-phosphogluconate and NADPH. The activity of CK was then determined from the rate of NADH formation measured at 340 nm.

### Total and differential cell count

Twenty microliters (µL) of blood were collected from the brachial plexus of anesthetized mice directly into a tube containing Turk's solution. Total cell counts were performed in a modified Neubauer chamber. A blood smear was prepared and stained with Giemsa and May-Grumwald stains. The percentage of lymphocytes, monocytes, and neutrophils were determined by counting at least 300 cells and using standard morphological criteria.

### Reduced glutathione (GSH)

Reduced glutathione (GSH) levels were measured using the method which quantifies the formation of 5,5′-dithiobis-(2-nitrobenzoic acid)- sulfidryl groups using a colorimetric assay. A sample of skeletal muscle tissue, quadriceps, (100 mg) was homogenized in 900 µL of 0.1 M phosphate buffer (pH 6.5), and the supernatant was added to 5,5′-dithiobis-(2-nitrobenzoic acid) to create the reaction mixture. The absorbance was measured at 412 nm in a microplate reader using GSH as an external standard [16].

### Intravital microscopy

Briefly, mice were anesthetized intraperitoneally (100 µl for each animal) with a mixture of ketamine (37.5 mg/ml, final concentration) and xylazine (2.5 mg/ml, final concentration). After the animals were anesthetized, the quadriceps muscle was exposed to visualize the vasculature. This muscle group was used because it is intensely exercised in the fatiguing exercise protocol and it has a large area which facilitates the capture of images from vessels. All vessels evaluated in this study were of a very specific diameter size (30–40 um). For all studies, we only used vessels, which were fully perfused at the time of data acquisition. Blood flow was inferred by the velocity of rhodamine labeled platelets and was found to be similar in both groups of animals. Throughout the experiment, the mouse was maintained at 37°C using a heating pad, and the exposed muscle was continuously perfused with PBS buffer. Leukocytes, fluorescently labeled by the intravenous administration of Rhodamine 6G-Sigma (0.5 mg/kg body weight), were observed using a microscope (Nikon, ECLIPSE 50i, 20x objective lens) outfitted with a fluorescent light source (epi-illumination at 510–560 nm, using a 590 nm emission filter). The numbers of rolling and adherent leukocytes were determined offline during the video playback analyses. Leukocytes were considered adherent to the venular endothelium if they remained stationary for at least 30s. Rolling leukocytes were defined as white cells moving at a velocity slower than that of the erythrocytes within a given vessel. To obtain the transmigration score, the quadriceps muscle of Lysm-eGFP mice was exposed, and the vasculature was stained by PE-coupled anti-PECAM-1 antibody (10 uL of a stock solution (0.2 mg/mL) was injected intravenously 2 minutes before imaging procedures. PE-coupled anti-CD31 was purchased from eBiosciences, clone 390). The neutrophil-endothelium interactions from postcapillary venules were recorded for twenty minutes using a confocal microscope (Olympus *UPRIGHT -* 488). The number of transmigrating neutrophils was determined offline during the video playback analyses. Briefly, the video recording was paused at 1-min time intervals, and the numbers of neutrophils inside and outside of the postcapillary venules were counted [17]. In each animal, two different fields were recorded and analyzed to determine the total number of neutrophils per field. The two values were averaged and 5–6 animals were used in each group. Data are representative of two independent experiments.

### Histology

A set of experiments was conducted to analyze histopathological parameters (fiber alignment and infiltration of inflammatory cells) in the quadriceps muscle. The skeletal muscle tissue was removed and fixed in buffered formaldehyde (10% in PBS) for 24 hours and routinely processed for paraffin embedding. Sections (5-µm thick) were obtained and stained with H & E [18]. The images were captured using a microscope at a resolution of 720×480 pixels (Cool SNAP-Procf Color, Media Cybernetics, Bethesda, MD) and evaluated using the program Image ProExpress version 4.0 for Windows (Media Cybernetics). These histopathological analyses were made by a professional pathologist.

### Real time - PCR

Expression of molecules of adhesion was quantified by Real-Time PCR as described previously [19]. Briefly, skeletal muscle tissue (quadriceps) was removed for analysis of transcript levels of E-selectin, L-selectin and PECAM. Total RNA was obtained using Trizol (Invitrogen, Carlsbad, CA) according to the procedure supplied by the manufacturer. Total RNA was reverse transcribed with SuperScript III (Invitrogen) as described by the manufacturer. Real-time quantitative PCR (qPCR) was performed in an ABI PRISM 7500 Fast detection system (Applied Biosystems, Carlsbad, CA) using SYBR Green PCR Master Mix (Applied Biosystems) with specific primer pairs. The relative expression level of genes was determined by the 2^−ΔΔCt^ method and data were normalized by 18S ribosome subunit expression levels. All reactions were replicated.

### Drug administration

In this study, 10 mg/kg of apocynin, a well-established NADPH oxidase inhibitor, and 3 mg/kg of superoxide dismutase (SOD) were administered 30 minutes before exercise protocol by intraperitoneal (i.p.) injection, to investigate the role of ROS in the neutrophil recruitment into quadriceps muscle. The control mice received PBS alone. SOD and apocynin drugs were administered in wild type C57Bl/6 and LysM-eGFP C57Bl/6 mice.

### Statistical analyses

All of the results are presented as the mean ± SEM. The data were analyzed by one-way analysis of variance and differences between groups were assessed using the Student-Newman-Keuls post-test. *P* values less than 0.05 were considered significant. The results are expressed as the means ± S.E.M.

## Results

### Fatiguing exercise protocol induced muscle injury and alteration in the numbers of circulating leukocytes

**Figure 2:**
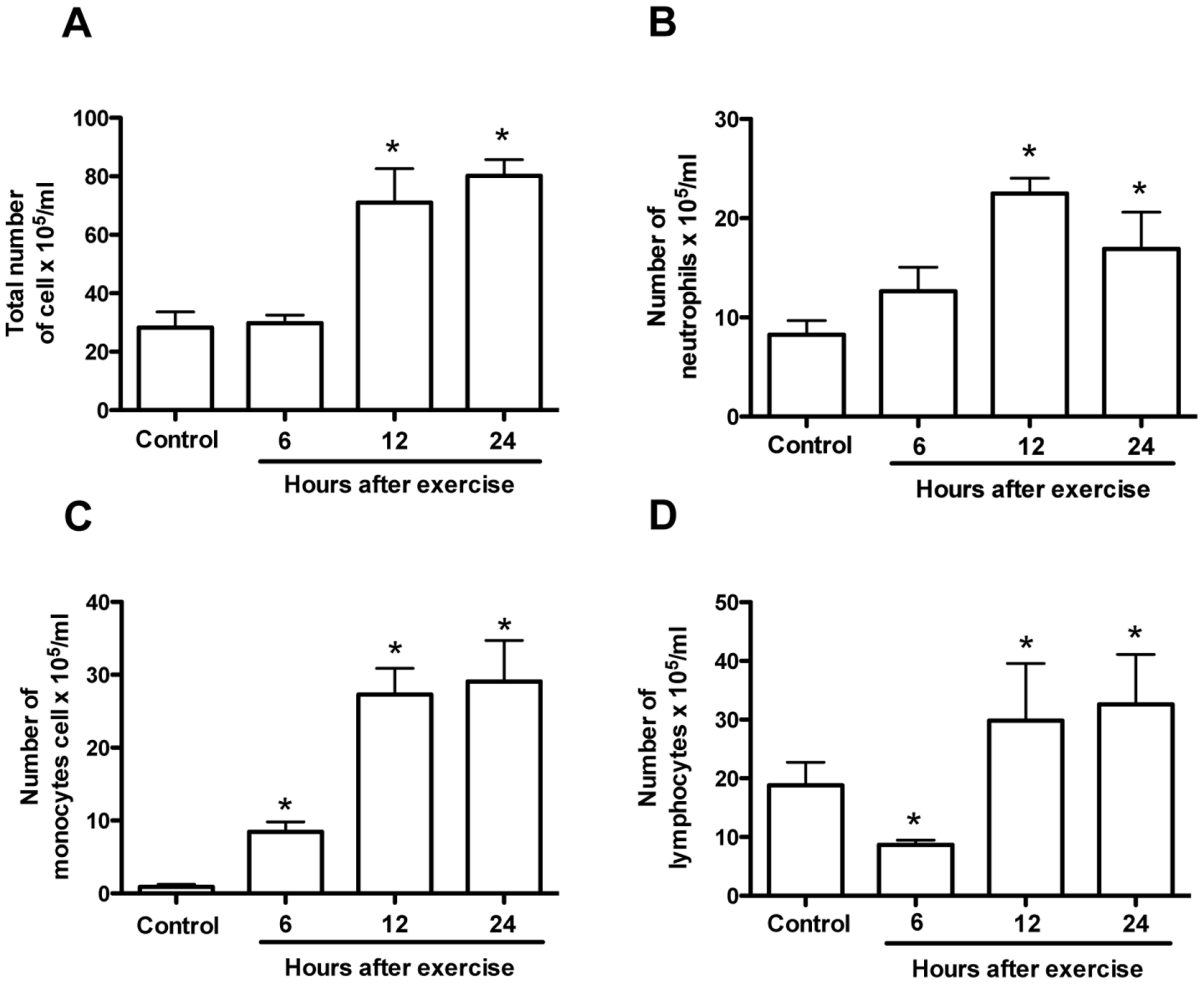
Number of leukocytes in the peripheral blood. Fatigue was induced by spontaneous running on an electric treadmill. At different time-points after the exercise protocol blood samples were collected and numbers of total cell (A) neutrophils (B), monocytes (C) and lymphocytes (D) were evaluated. In this study WT C57Bl/6 mice were used. The results are presented as the mean ± SEM (n = 4–6). *P<0.05 when compared with the control group.

During the exercise protocol the mice reached a maximum speed of 15.8±3.7 m/min, which resulted in a total time of 56.3±6.7 minutes and a total distance of 438.6±68.4 meters. Fatiguing exercise protocol induced a rapid increase in oxygen consumption (VO2) that indicates an enhancement of metabolic rate exercise-induced (Fig. 1B). The plasma lactate concentration, an important marker for exercise intensity, was analyzed before and immediately after exercise. The exercise protocol was able to increase the peripheral blood lactate concentration (Fig. 1C). The serum CK activity, a specific muscle injury marker, was also elevated at the 6 and 12 hours post-exercise (Fig. 1D) and the glucose level, that is an energetic substrate, was reduced in blood immediately after the fatiguing exercise protocol (Fig. 1E). These results indicate that our exercise protocol was at a high intensity level and induces skeletal muscle damage. Furthermore, this treadmill exercise protocol increased the number of leukocytes in the peripheral blood at 12 and 24 hours post-exercise (Fig. 2A) associated with an increase in the numbers of neutrophils and monocytes at the same time points (Fig. 2B and C). Interestingly, the number of lymphocytes was reduced at 6 hours post-exercise and then increased 12 and 24 hours after exercise (Fig. 2D). Next, to verify whether leukocyte-endothelium interactions were dependent on muscle injury, we applied different exercise protocols. First, mice performed an exercise running at 60% of duration, but the same velocity of the fatiguing exercise protocol. Next, mice performed an exercise running at 60% of intensity, but for the same period of time of the fatiguing exercise protocol. Therefore, in both protocols mice did not exercise until fatigue. Consistently, there was a lower increase of CK activity in both cases showing mild muscle injury but the same number of rolling and adherent cells when compared with fatigued group (Figure S1).

### Fatiguing exercise protocol induced neutrophil accumulation in quadriceps muscle

**Figure 3:**
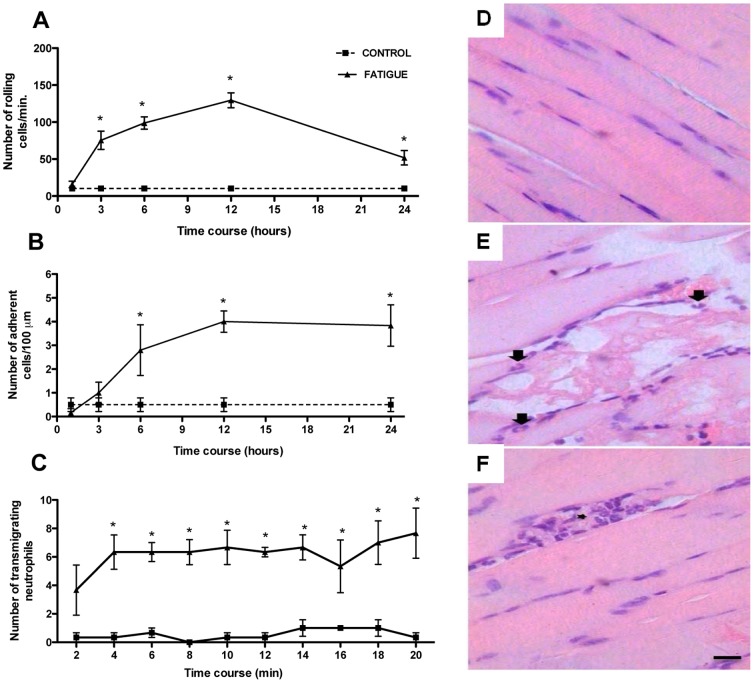
Recruitment of neutrophil into quadriceps muscle. Fatigue was induced by spontaneous running on an electric treadmill. At different time-points after the exercise protocol, mice were anesthetized and muscle postcapillary venules selected for counting numbers of rolling (A), adherent (B) and transmigrating (C) leukocytes using epifluorescence and confocal intravital microscopy, respectively. The time course on the x-axis in the 3C figure represents the time of recording. It was recorded for 20 minutes. After intravital microscopy, quadriceps muscle samples were removed for histological analyses. Tissue section from control (D) and 12 hours after the exercise (E–F) mice are shown for illustration. Neutrophil infiltration is shown by arrows and inflammatory foci consisting predominantly of neutrophils are shown by asterisks. Original scale bars 50 µm for all panels. In this study WT C57Bl/6 and LysM-eGFP C57Bl/6 mice were used. The results are presented as the mean ± SEM (n = 5). *P<0.05 when compared with the control group.

**Figure 4:**
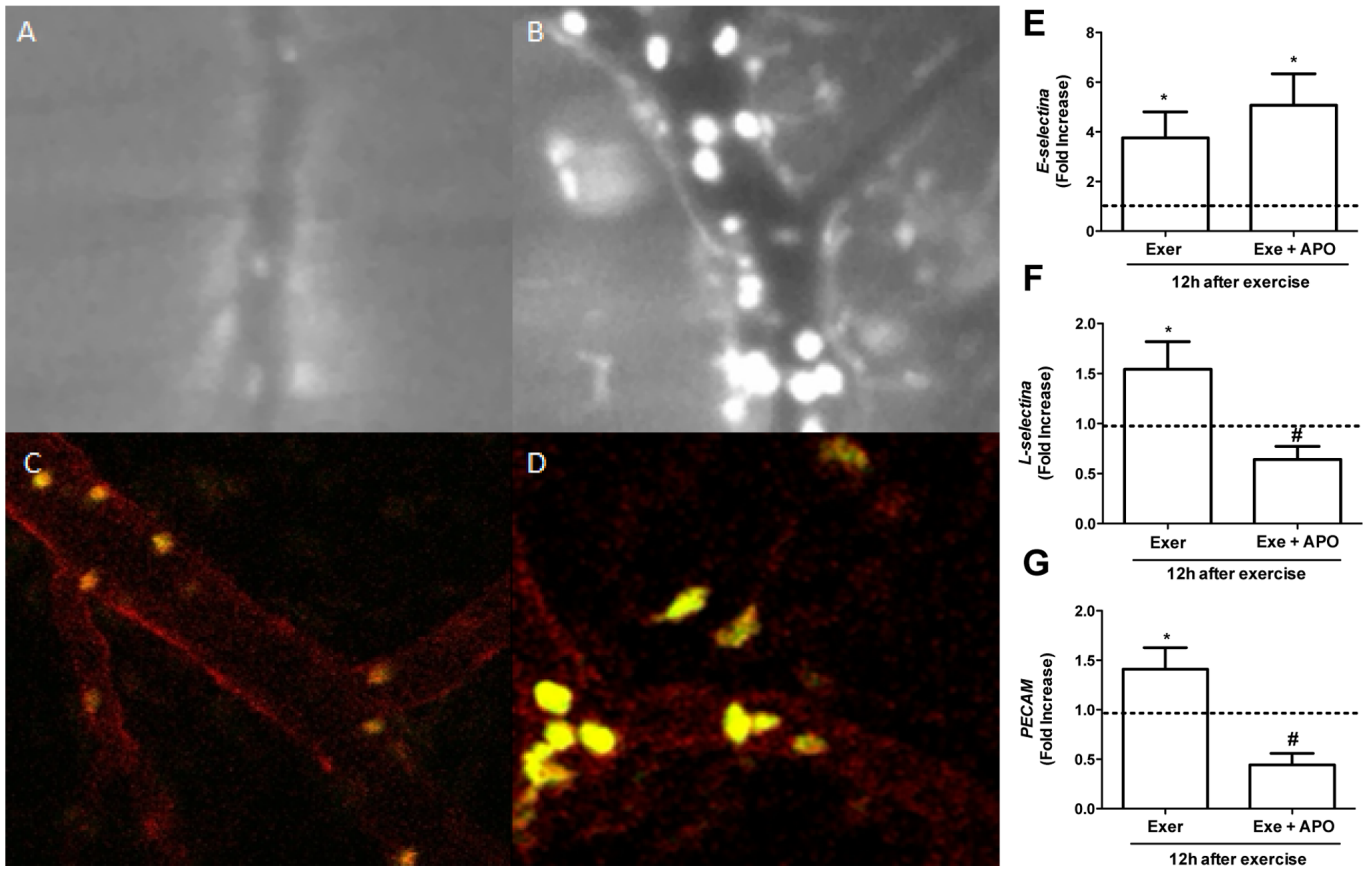
Leukocytes-endothelium interaction in quadriceps muscle after exercise and adhesion molecules expression. Fatigue was induced by spontaneous running on an electric treadmill. Quadriceps muscle was exposed to visualize the vasculature 12(A) and exercised wild-type mice (B). Confocal intravital microscopy images are shown in control (C) and exercised LysM-eGFP mice (D). Using qPCR technique, the molecules expression was quantified: E-selectin (E), L-selectin (F) and PECAM (G) were evaluated 12 hours after the exercise. APO  =  apocynin and dotted line represents the control group. The results are presented as the fold increase (n = 4, control group and n = 6, exercised group). *P<0.05 when compared with the control group.

To examine whether treadmill exercise promotes leukocyte migration into muscle tissue, we performed intravital microscopy of the post-capillary venules in the quadriceps muscles of exercised animals until fatigue. First, using epifluorescence intravital microscopy, we observed an increase in the numbers of rolling and adherent leukocytes from 3 to 24 hours after running (Fig.3A–B and Fig.4A–B and video S1–S2). Then, histological analyses of quadriceps muscle sections were evaluated 12 hours after the fatiguing exercise protocol. This time-point was chosen because it was the peak of the inflammatory response. The quadriceps muscle from the exercise group displayed disruption of fiber alignment and partial destruction of the muscle fibers associated with a predominant neutrophilic infiltration. Representative pictures are shown in Figure 3D–F. These results are consistent with the intravital microscopy data. Then, using LysM-eGFP mice, we found that the predominant numbers of rolling and adherent leukocytes were neutrophils (data not shown). Furthermore, by confocal intravital microscopy, we showed an increase of transmigrating neutrophils into the parenchymal tissue muscle after exercise when compared with the control mice at rest (Fig.3C and Fig.4C–D and video S3–S4).

In order to evaluate if the fatiguing exercise protocol could induce expression of adhesion molecules, we investigated the expression of mRNAs for E-selectin, L-selectin and PECAM. These molecules were chosen because they may play a role in mediating rolling, adhesion and transmigrating of neutrophils in other systems [20]. The results of quantitative PCR showed that E-selectin (Fig. 4E) expression increased after 12 hours. Treatment with apocynin, an NADPH oxidase inhibitor, did not alter E-selectin expression. L-selectin expression also increased after 12 hours, but apocynin administration blocked this elevation (Fig. 4F). Similar results were observed at the level of PECAM expression; ie. exercise induced an elevation of gene expression which was reduced by apocynin treatment (Fig. 4G). These results suggest that exercise increased the recruitment of leukocytes and induced an increase in the expression of adhesion molecules in tissue. Expression of these molecules was inhibited by apocynin treatment, suggesting that ROS were involved in this process. This study also evaluated the expression of ICAM-1 and VCAM-1 molecules by qPCR and could not find any difference in cell adhesion molecule expression between control and exercised groups at 12 hour after exercise (data not shown).

### ROS play a relevant role to leukocyte/endothelial cell interactions in muscle tissue after the fatiguing exercise

**Figure 5:**
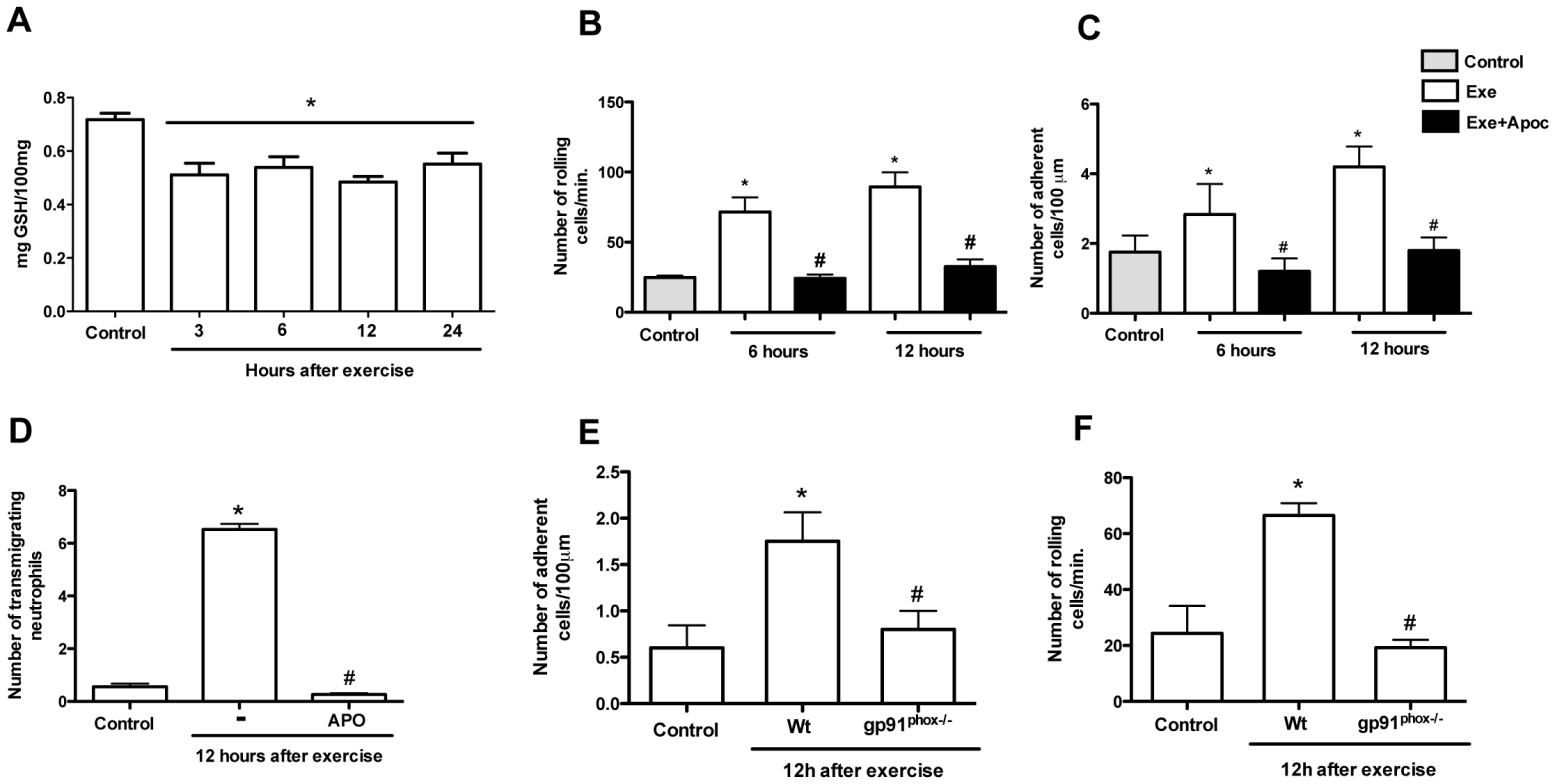
ROS production from NADPH oxidase on leukocyte recruitment. Fatigue was induced by spontaneous running on an electric treadmill. GSH levels were measured at different time-points after exercise protocol (A). Mice received an injection of apocynin (10 mg/kg, i.p.) 30 minutes before exercise and numbers of rolling (B), adherent (C) and transmigrating (D) leukocytes was evaluated 6 and 12 hours after the exercise. In another experimental setting, numbers of rolling (E) and adherent (F) leukocytes was evaluated 12 hours after the exercise using mutant mice (gp91^phox-/-^). The results are presented as the mean ± SEM (n = 5). *P<0.05 when compared with the control and # when compared with exercised mice.

**Figure 6:**
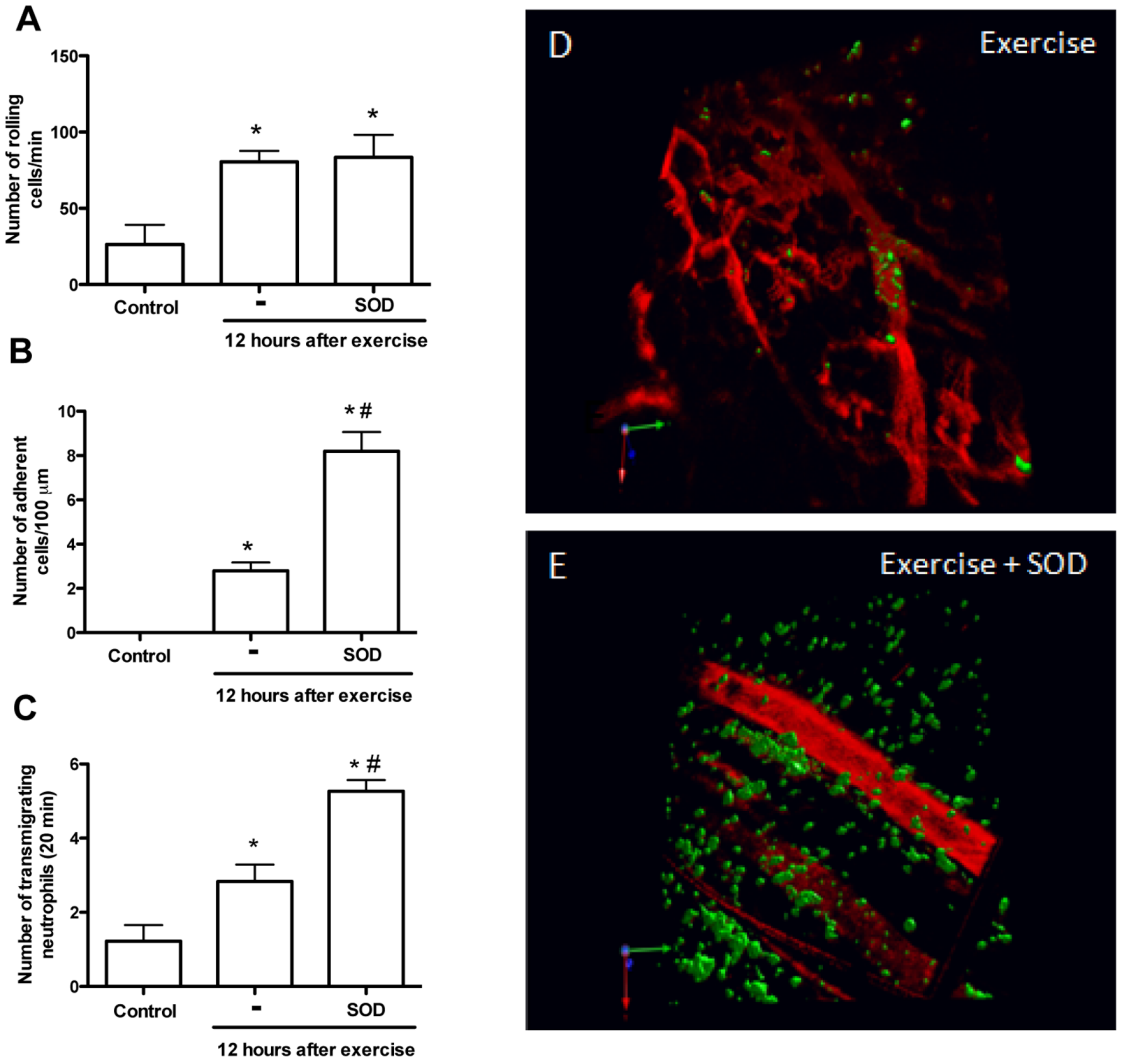
Superoxide dismutase (SOD) exacerbated adhesion and transmigration of neutrophil. Fatigue was induced by spontaneous running on an electric treadmill. Superoxide dismutase (3 mg/kg, i.p.) was injected 30 minutes before exercise. Using LysM-eGFP mice, numbers of rolling (A), adherent (B) and transmigrating (C) neutrophil was evaluated 12 hours after the exercise. These results were confirmed by 3D images from exercised (D) and exercised + SOD (E) mice. The results are presented as the mean ± SEM (n = 4, control group and n = 6, exercised and exercised + SOD group). *P<0.05 when compared with the control and # when compared with exercised mice. (red  =  vessels; green  =  neutrophils).

Once we established that neutrophil accumulation occurred in skeletal muscle tissue after the fatiguing exercise, we determined the role of ROS in leukocyte-endothelium interaction. First, we determined GSH levels in mice after the fatiguing exercise protocol. There was a reduction in the amount of GSH at different time points which indicates that ROS increased after exercise (Fig. 5A). We then evaluated the role of endogenous ROS for neutrophil accumulation in muscle after exercise. The mice were treated with a single dose of apocynin 30 minutes before the fatiguing exercise protocol was initiated. In animals treated with apocynin there was less neutrophil rolling, adhesion and transmigration (Fig.5A–D). Moreover, the gp91^phox^ deficiency also reduced the numbers of rolling (Fig. 5E) and adherent (Fig. 5F) leukocytes 12 hours after the exercise. The administration of SOD (superoxide dismutase), the enzyme responsible for converting radical anion superoxide (O·^−^) into hydrogen peroxide (H_2_O_2_), increased the numbers of adherent cells (Fig. 6B) and transmigrating neutrophils at the same time-points (Fig. 6C). However, SOD treatment did not alter leukocyte rolling (Fig. 6A). The treatment with SOD alone did not cause any alteration in the number of total circulating leukocytes and did not alter the number of rolling and adherent cells (data not shown).

## Discussion

Notable results from this study are as follows: (1) this was the first study of its kind to show interaction of neutrophils with endothelial cells in quadriceps muscle by intravital microscopy after a fatiguing exercise protocol; (2) we observed clear rolling, adhesion and subsequent transmigration of neutrophils induced by exercise. This is consistent with other studies demonstrating leukocyte accumulation in tissue as determined by histology [1], [20]; (3) leukocyte-endothelial cell interactions were accompanied by increased ROS production as identified by evaluation of the depletion of GSH in exercised muscles; (4) reduction of ROS production, as observed in gp91^phox^-deficient mice or by pharmacological blockade of NADPH oxidase with apocynin, prevented leukocyte/endothelial cell interactions; (5) in contrast, treatment with SOD, which enhances the production of hydrogen peroxide, enhanced neutrophil transmigration in mice. Altogether, these results uniquely show that intense exercise is capable of causing neutrophil-endothelial cell interactions in the microvasculature of exercised muscles in a ROS-dependent manner.

As expected, the fatiguing exercise protocol changed some physiological responses, including lactate level and oxygen consumption. After exercise, there was an increase in the number of circulating neutrophils and monocytes. Changes in neutrophil counts after exercise appear to be associated with the mobilization from the marginal pool and bone morrow [21]. Total lymphocyte counts significantly decreased followed by an increase after the fatiguing exercise protocol. This biphasic response in the number of lymphocytes after an acute bout of endurance exercise has been observed previously and could be explained by the “open window theory” [21]. The importance of the suppression in lymphocyte number after fatiguing exercise is not entirely known, but may contribute to facilitate infections. Changes in lymphocyte number appear to be secondary to the increase in circulating levels of stress hormones, including epinephrine, norepinephrine and cortisol. It has been suggested that catecholamines induce the increase in lymphocyte numbers, whereas cortisol induces lymphopenia after exercise [21]. Transient changes of leukocyte number in the circulation may also reflect hormonal changes (adrenergic and cortisol) induced by exercise and it is of unknown significance [21], [22]. Whether persistent changes of lymphocyte numbers by repeated intense exercise may be of sufficient intensity to cause suppression of immunological responses is still an open question and was not investigated here.

In our experiments, after the fatiguing exercise protocol, lactate and CK activity were increased in plasma and this increase was associated with disruption of the fiber alignment and partial destruction of the muscle fibers, as determined by histology. In the last decade, it has become clear that acute exercise can induce significant changes in skeletal muscle [1], [2], [4], [20], [22]. These changes are characterized by ultra structural loss of muscle architecture, increase of muscle proteins in the bloodstream including creatine kinase (CK) and myoglobin (Mb), loss of muscular strength and a range of movement and muscle pain [2]–[4].

Exercise-induced muscle damage may be associated with a local inflammatory response involving leukocyte accumulation in damaged muscle tissue [3],[23],[24]. Different methods have been used to detect leukocytes accumulation into skeletal muscle tissue, including myeloperoxidase (MPO) biochemical assay, antibody staining procedure (immunohistochemistry or immunofluorescence) and white blood cell (WBC) radionuclide imaging [20], [23], [24]. However, leukocyte influx is not detected by these methods in all studies and there has been much controversy about the real extent of leukocyte influx in the muscle after acute exercise.

To directly observe leukocyte influx and to gain insights into possible mechanisms involved in leukocyte recruitment after exercise, we assessed leukocyte-endothelial interactions within the muscular microvasculature using intravital microscopy. This is a robust technique capable of visualizing biological phenomena in living organisms. Several studies have shown leukocyte and endothelial cell interactions in the cremaster muscle after various inflammatory stimuli [16], [25] but this has been applied to exercise for the first time in the present study. We demonstrated that neutrophils interact with endothelial cells and then transmigrate to the muscle tissue following the fatiguing exercise protocol. Therefore, these results clearly show that a physiological stimulus (exercise) induces leukocyte influx, a hallmark of inflammation, in the muscle, concurring with the idea that inflammation is a physiological host response.

It remains elusive whether infiltrating neutrophils contribute to skeletal muscle damage or to skeletal muscle tissue repair [1]. There is some evidence that neutrophils induce the production of pro-inflammatory molecules that may contribute to the remodeling and repair process by activating satellite cells and liberating angiogenic factors [1], [3], [25]. Indeed, we have shown in preliminary studies that leukocyte-endothelium interaction also occurs after exercise at lower intensity when tissue injury is mild (Figure S1). The relevance of leukocyte influx into the quadriceps muscle in our model is not known yet and clearly deserves further investigation. It has been suggested, however, that exercise-associated inflammation may be relevant for muscle remodeling and adaptation to the new load of exercise. This remodeling process could occur by increasing in the hypertrophic signaling pathway of the skeletal muscle cell or by inducing angiogenesis in this tissue [1], [26]. We are currently investigating these possibilities in our model.

The present study also investigated the role played by ROS for the neutrophil migration into muscle tissue resulting from treadmill exercise in mice. Our results show that vigorous exercise triggered ROS production in skeletal muscle, as assessed by a significant decrease in tissue levels of GSH. It has been shown that the oxidative stress may be important for the inflammatory response after skeletal muscle injury induced by trauma [27]. Our results concur with the latter findings. We have used two distinct strategies to assess the role of ROS for the recruitment of leukocytes into the skeletal muscle tissue, a pharmacological strategy (apocynin) and genetically modified animals (gp91^phox-/-^). Our results show that the drug or genetic deletion of NADPH-oxidase inhibited rolling, adhesion and transmigration of leukocytes. We have not determined the source of ROS in this system but ROS may be produced by endothelial cells [28], [29], skeletal muscle cells [10], [11], [30] and leukocytes [31], because these cells express NOX-2, the NADPH-oxidase which contains the gp91^phox^ subunit [32], [33]. The results also showed enhancement of the numbers of leukocytes adherent and transmigrating when superoxide dismutase (SOD) is used, suggesting that hydrogen peroxide (H_2_O_2_) is the probable ROS molecule involved. In support of this possibility, H_2_O_2_ produced endogenously induced upregulation of adhesion molecules and chemokines and was responsible for increased numbers of rolling and adherent leukocytes [8]. Therefore, we show for the first time that ROS, possibly H_2_O_2_, from the NOX-2 NADPH-oxidase plays an important role in the recruitment of leukocytes to the muscle after exercise *in vivo*.

Here, we show that the fatiguing exercise protocol can increase the expression of E-selectin, L-selectin and PECAM that could explain the rise in the numbers of rolling and adherent leukocytes 12 hours after the fatiguing exercise protocol. These results also show that reduction of ROS production by apocynin can modulate this response. This is consistent with studies showing that ROS can induce an increase in gene expression of cell adhesion molecules in pulmonary disease models [34]. Thus, ROS may regulate cell recruitment to the exercised muscle via modulation of leukocyte adhesiveness to the vessel wall. The precise molecules which mediate leukocyte influx in the system have not been investigated here and will be the subject of future studies. One potential function of ROS may be to recruit cells to the exercised muscle and orchestrate the resolution of the function of the damaged tissue and induce the proper adaptive response to exercise training. ROS–mediated infiltration of neutrophils after exercise may also contribute to the exercise remodeling processes, including angiogenesis, hypertrophic response and mitochondrial biogenesis [35]. In unpublished data, we could see that after 6 weeks of running training, skeletal muscle (quadriceps) presents over expression of E-selectin, L-selectin, PECAM. The training period also increased the expression of Pax3, that is an important hypertrophic factor produced by satellite cells and apocynin administration during the training period inhibited this response. Taken together, our data suggest that exercise-triggered ROS production enhanced leukocyte-endothelium interaction, which coordinates neutrophil infiltration to the exercised skeletal muscle.

## Supporting Information

Figure S1 **The leukocytes recruitment also occurs in a lower level of exercise load.** Exercise protocol was reduced to 60% of maximal velocity and 60% of maximal duration. Immediately and 12 after exercise **b**lood samples were collected for measurement of lactate (A) and CK (B), respectively. The number of rolling (C) and adherent (D) was evaluated 12h after exercise. The results are presented as the mean ± SEM (n = 4–6). The results are presented as the mean ± SEM (n = 5). *P<0.05 when compared with the control and # when compared with exercised mice.
(TIF)

Video S1 **Epifluorescence intravital microscopy microscopy images from postcapillary venule of control group (non-exercised):** Leukocytes, fluorescently labeled by the i.v. administration of Rhodamine (6G) were observed using a microscope with a fluorescent light source. The number of rolling and adherent leukocytes was determined offline during the video playback analyses. Leukocytes were considered adherent to the venular endothelium if they remained stationary for at least 30s. Rolling leukocytes were defined as white cells moving at a velocity slower than that of the erythrocytes within a given vessel. Representative video showing non-exercised animal.
(WMV)

Video S2 **Epifluorescence intravital microscopy microscopy images from postcapillary venule of fatigued group.** Experiments were performed as described in ‘Video S1’. This group received the fatigue protocol exercise and then 12 hours later these images were captured. Representative video showing exercised animal.
(WMV)

Video S3 **Confocal intravital microscopy images from postcapillary venule of control group (non-exercised):** Lysm-eGFP mice were anesthetized by intraperitoneal injection to obtain transmigration score was. The quadriceps muscle was exposed, and the vasculature was stained by PE-coupled anti-PECAM-1 antibody. Neutrophil-endothelium interactions within muscle microvasculature were recorded for twenty minutes using a confocal microscope. The number of transmigrating neutrophils was determined offline during the video playback analyses. Briefly, the video recording was paused at 1-min time intervals, and the numbers of neutrophils inside and outside of the postcapillary venules were counted. Representative video showing non-exercised animal.
(WMV)

Video S4 **Intravital confocal microscopy images from postcapillary venule of control group (non-exercised):** Experiments were performed as described in ‘Video S1’. This group received the fatigue protocol exercise and then 12 hours later these images were captured. Representative video showing exercised animal.
(WMV)
