# Supplementary figures and images for: Treadmill Exercise Induces Neutrophil Recruitment into Muscle Tissue in a Reactive Oxygen Species-Dependent Manner. An Intravital Microscopy Study
Source: PLoS One. 2014 May 5;9(5):e96464. doi: 10.1371/journal.pone.0096464 (PMC4010495; doi:10.1371/journal.pone.0096464)

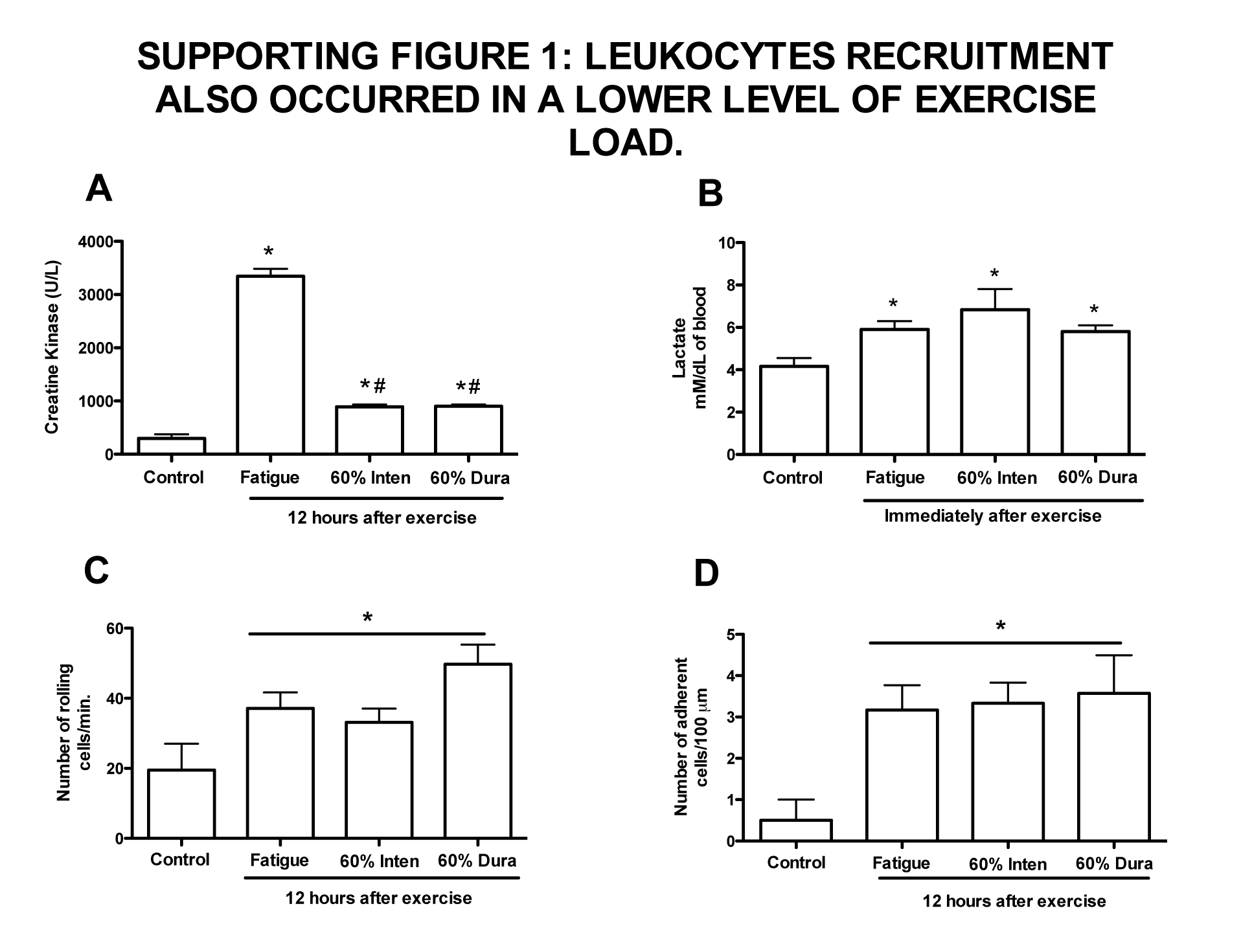

Supplement: Figure S1 — The leukocytes recruitment also occurs in a lower level of exercise load. Exercise protocol was reduced to 60% of maximal velocity and 60% of maximal duration. Immediately and 12 after exercise blood samples were collected for measurement of lactate (A) and CK (B), respectively. The number of rolling (C) and adherent (D) was evaluated 12h after exercise. The results are presented as the mean ± SEM (n = 4–6). The results are presented as the mean ± SEM (n = 5). *P<0.05 when compared with the control and # when compared with exercised mice. (TIF) [file pone.0096464.s001.tif]
